# Improved Prognostic Stratification Using Circulating Tumor Cell Clusters in Patients with Metastatic Castration-Resistant Prostate Cancer

**DOI:** 10.3390/cancers13020268

**Published:** 2021-01-13

**Authors:** Chun Wang, Zhenchao Zhang, Weelic Chong, Rui Luo, Ronald E. Myers, Jian Gu, Jianqing Lin, Qiang Wei, Bingshan Li, Timothy R. Rebbeck, Grace Lu-Yao, William K. Kelly, Hushan Yang

**Affiliations:** 1 Department of Medical Oncology, Sidney Kimmel Cancer Center, Thomas Jefferson University, Philadelphia, PA 19107, USA; chun.wang@jefferson.edu (C.W.); zhenchao.zhang@jefferson.edu (Z.Z.); weelic.chong@jefferson.edu (W.C.); rui.luo@jefferson.edu (R.L.); Ronald.Myers@jefferson.edu (R.E.M.); Grace.LuYao@jefferson.edu (G.L.-Y.); William.Kelly@jefferson.edu (W.K.K.); 2 Department of Epidemiology, MD Anderson Cancer Center, Houston, TX 77030, USA; jian.gu@mdanderson.org; 3 Department of Medicine, GW Cancer Center, George Washington University, Washington, DC 20037, USA; jilin@mfa.gwu.edu; 4 Department of Molecular Physiology and Biophysics, Vanderbilt University, Nashville, TN 37235, USA; qing.wei@vanderbilt.edu (Q.W.); bingshan.li@vanderbilt.edu (B.L.); 5 Department of Medical Oncology, Dana-Farber Cancer Institute, Boston, MA 02115, USA; Timothy_Rebbeck@dfci.harvard.edu; 6 Department of Epidemiology, Harvard T.H. Chan School of Public Health, Boston, MA 02115, USA

**Keywords:** circulating tumor cell, circulating tumor cell cluster, metastatic castration-resistant prostate cancer, progression-free survival, overall survival

## Abstract

**Simple Summary:**

Metastatic castration-resistant prostate cancer (mCRPC) is the most aggressive and deadly form of prostate cancer. As a bone-predominant metastatic disease, liquid biopsy-based biomarkers have advantages in monitoring cancer dynamics. Previous studies have demonstrated the associations between circulating tumor cells (CTCs) and mCRPC outcomes, but little is known about the prognostic value of CTC-clusters. In this study, we investigated the associations of CTCs and CTC-clusters with mCRPC prognosis, individually and jointly, using longitudinal samples. We confirmed the associations of CTC counts with mCRPC outcomes in both baseline and longitudinal analyses. Our results also showed that the presence of CTC-clusters alone had prognostic value and that CTC-clusters may further improve CTC-based prognostic stratification in mCRPC. Our findings suggest the potential of combing CTC and CTC-clusters as non-invasive means to monitor progression and predict survival in mCRPC and build a premise for in-depth genomic and molecular analyses of CTCs and CTC-clusters.

**Abstract:**

Liquid biopsy-based biomarkers have advantages in monitoring the dynamics of metastatic castration-resistant prostate cancer (mCRPC), a bone-predominant metastatic disease. Previous studies have demonstrated associations between circulating tumor cells (CTCs) and clinical outcomes of mCRPC patients, but little is known about the prognostic value of CTC-clusters. In 227 longitudinally collected blood samples from 64 mCRPC patients, CTCs and CTC-clusters were enumerated using the CellSearch platform. The associations of CTC and CTC-cluster counts with progression-free survival (PFS) and overall survival (OS), individually and jointly, were evaluated by Cox models. CTCs and CTC-clusters were detected in 24 (37.5%) and 8 (12.5%) of 64 baseline samples, and in 119 (52.4%) and 27 (11.9%) of 227 longitudinal samples, respectively. CTC counts were associated with both PFS and OS, but CTC-clusters were only independently associated with an increased risk of death. Among patients with unfavorable CTCs (≥5), the presence of CTC-clusters signified a worse survival (log-rank *p* = 0.0185). mCRPC patients with both unfavorable CTCs and CTC-clusters had the highest risk for death (adjusted hazard ratio 19.84, *p* = 0.0072), as compared to those with <5 CTCs. Analyses using longitudinal data yielded similar results. In conclusion, CTC-clusters provided additional prognostic information for further stratifying death risk among patients with unfavorable CTCs.

## 1. Introduction

Prostate cancer (PCa) is the most commonly diagnosed cancer and the second leading cause of cancer-related death among men in the United States [1]. In patients who are diagnosed with or progress to advanced or metastatic PCa, the standard treatment is androgen deprivation therapy (ADT). Initial responses in patients receiving ADT are generally favorable, but almost all patients ultimately progress to metastatic castration-resistant prostate cancer (mCRPC) [2,3]. Approximately 10–20% of PCa patients develop CRPC within five years of diagnosis, and 84% of newly diagnosed CRPC have metastases [3,4]. The all-cause mortality for PCa is estimated to be 219,360 in 2020, and mCRPC accounts for 19.5% of these deaths [5].

Remarkable progress has been made in the use of tissue-based molecular analyses (i.e., precision genomics) to guide treatment decisions of many cancers. However, such tissue-based genomic profiling is challenging in mCRPC due to several reasons. First, mCRPC is a bone-predominant metastatic disease, thus tissue samples are not always obtainable. Second, the yield of tumor tissues from metastatic sites can often be quite low, particularly when sampling from bone metastases [6]. Third, due to intratumoral heterogeneity [6,7], biopsy samples may not fully represent the tumor, and sequencing results could yield inaccurate findings. Fourth, during the course of disease progression and treatment, genomic signatures change over time to evade therapeutic or immune attacks [8,9], but the invasive nature of tumor tissue biopsy makes it infeasible to perform repeated biopsies to guide each new treatment decision over time. Therefore, non-invasive biomarkers based on liquid biopsy samples, such as circulating tumor cells (CTCs), need to be developed to guide mCRPC treatment decisions and monitor treatment response or resistance.

CTCs are shed from primary or metastatic tumors into the blood and have extremely high malignant potential. Since CTCs constitute “seed cells” for metastasis, they are arguably the most important subset of tumor cells to monitor and treat [10,11]. Unlike tissues or biopsies, CTCs can be repeatedly, non-invasively measured, and characterized in a real-time manner. A multicenter prospective landmark study by de Bono et al. [12] reported that the CTC number at different time points after treatment was the strongest independent predictor of overall survival (OS) in mCRPC, which led to the expansion of the clinical utilization of the CellSearchTM system, the FDA-cleared method for CTC enumeration, from breast cancer to prostate cancer. Numerous subsequent studies further confirmed the prognostic value of CTC enumeration in mCRPC [13,14,15,16,17,18]. A pooled analysis of five randomized phase III clinical trials also suggested using the baseline CTC and its conversion (≥5 CTCs at baseline and <5 CTCs at follow-up visit) as response endpoints for early-phase mCRPC clinical trials, as these exhibited the highest discriminatory power among the indices tested [19].

In addition to disseminating as individual cells, tumor cells also collectively migrate as clusters, in which cell-cell adhesion remains intact [20,21]. Clustered CTCs in peripheral blood have been reported in patients with different cancer types, but at lower frequencies than single CTCs, whereas within primary lesions of epithelial tumors, collective migration is more prominent than single-cell motility [21]. Several recent studies, including ours, reported a worse prognosis associated with CTC-clusters in patients with breast cancer [22,23,24] and lung cancer [25]. The seminal study by Aceto et al. [22] found that the presence of CTC-clusters was associated with shorter overall survival (OS) in patients with PCa. CTC-clusters were also identified in patients with mCRPC, but very few studies have evaluated the prognostic value of CTC-clusters and these limited reports showed inconsistent findings [26,27].

As yet, no study has been reported to evaluate whether CTC-clusters can further improve CTC-based prognostic stratification in mCRPC. Furthermore, no in-depth analysis has been conducted to explore the prognostic value of CTC-clusters, by using longitudinally collected data. Herein, based on an ongoing mCRPC cohort with longitudinal samples, we conducted, to our best knowledge, the first study that evaluated the prognostic value CTC-clusters in high-risk mCRPC patients with high CTC levels.

## 2. Results

### 2.1. Patient Characteristics

A total of 64 mCRPC patients were included in this analysis (Table 1). At the first time of biomarker measurement (baseline), 18 (28.1%) patients had newly diagnosed mCRPC and the median age was 71.8 (range 53.0–93.0) years old. Among these patients, 49 (76.6%) patients were whites, 53 (82.8%) patients had an Eastern Cooperative Oncology Group (ECOG) performance score of 0–1, 60 (93.8%) had bone metastasis, and 13 (20.3%) had visceral metastasis (e.g., liver, lung). Of the 64 patients, 35 (54.7%) had received androgen receptor signaling inhibitors (ARSi) (e.g., enzalutamide, abiraterone acetate) and 10 (15.6%) had received chemotherapy from metastatic diagnose to the baseline sample collection. There were 44 (68.8%) patients treated by ARSi and 16 (25%) patients treated by cytotoxic reagents after the baseline blood draw. The baseline (median) laboratory results were as follows: prostate-specific antigen (PSA) = 9.5 ng/mL, hemoglobin (HGB) = 12.0 g/dL, alkaline phosphatase (ALP) = 88.5 IU/L, albumin (ALB) = 4.1 g/dL, and lactate dehydrogenase (LDH) = 216 IU/L. During a median follow-up of 14.4 months (interquartile range: 9.7–19.1 months), 45 (70.3%) patients had progressive disease and 23 (35.9%) died.

### 2.2. Associations between Baseline CTCs and Clinical Outcomes

**Figure 1:**
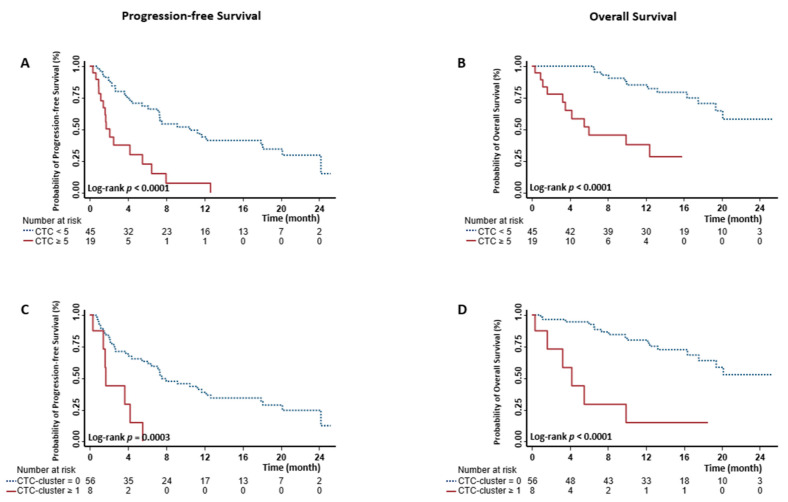
Kaplan-Meier estimates of the probability of progression-free survival (PFS) and overall survival (OS) in metastatic castration-resistant prostate cancer (mCRPC). Survival differences were compared between patients with <5 circulating tumor cells (CTCs) and ≥5 CTCs ((**A**) for PFS and (**B**) for OS), or between those with 0 and ≥1 CTC-clusters ((**C**) for PFS and (**D**) for OS).

We first evaluated the prognostic values of baseline CTCs and CTC-clusters individually. CTC enumeration results (Table S1) showed that CTCs were detected in 24 (37.5%) patients, and among them, 19 had ≥5 CTCs. We then categorized mCRPC patients as having either unfavorable (≥5 CTC/7.5 mL) or favorable (<5 CTC/7.5 mL) CTC counts as described previously [12]. The patients with unfavorable CTC counts had significantly shorter survival time, compared to those with favorable CTC counts (median survival time 2.1 vs. 10.5 months, log-rank *p* < 0.0001 for progression-free survival (PFS); 6.0 months vs. not reached, log-rank *p* < 0.0001 for OS) (Figure 1A,B and Table 2). mCRPC patients with unfavorable CTC counts had a 4.03-fold (hazard ratio (HR) 4.03, 95% confidence interval (CI) 2.09–7.77) increased risk for developing the progressive disease and a 6.90-fold (HR 6.90, 95% CI 2.72–17.47) increased risk for death (Table 2). When conducting univariate analyses on each demographic, clinical, and laboratory variable, we found an association between outcomes and performance status, previous chemotherapy, ARSi, and cytotoxic therapy after the baseline blood draw, PSA, HGB, ALP, and ALB (Table S2). We then included these covariates into the multivariate Cox model. After adjustment, the associations between CTC counts and clinical outcomes remained significant (*p* = 0.0101 for PFS and *p* = 0.0134 for OS, Table 2).

### 2.3. Associations between Baseline CTC-Clusters and Clinical Outcomes

CTC-clusters were identified in 8 (12.5%) of 64 patients. Representative immunofluorescent images of CTC-clusters are shown in Figure S1. Except for one patient, CTC-clusters were mostly observed in patients with ≥5 CTCs, which was similar to our findings in breast cancer [23]. Among 5 (62.5%) of the 8 patients with CTC-clusters, had clusters each consisting of 2 cells (Table S1). We then stratified mCRPC patients by whether or not they had CTC-clusters. The patients with ≥1 CTC-clusters had poor survival compared to those without any CTC-cluster (median survival time 1.7 vs. 7.5 months, log-rank *p* = 0.0003 for PFS; 4.2 months vs. not reached, log-rank *p* < 0.0001 for OS) (Figure 1C,D and Table 2). mCRPC patients with CTC-clusters had a 4.33-fold (HR 4.33, 95% CI 1.81–10.37) increased risk for progression and a 6.58-fold (HR 6.58, 95% CI 2.50–17.33) increased risk for death (Table 2). After adjusting covariates, only the associations of CTC-clusters with OS remained significant (*p* = 0.0299) (Table 2).

### 2.4. Prognostic Stratification Using Baseline CTCs and CTC-Clusters

We were interested in learning whether CTC-clusters could further stratify prognostic risk in patients with unfavorable CTC counts. To this end, we categorized patients into three risk groups. The low-risk group (*n* = 44) included those with favorable CTC counts (<5 CTCs), and no patients in this group had a CTC-cluster. The medium-risk group (*n* = 12) included those with unfavorable CTC counts (≥5 CTCs) but without a CTC-cluster. The high-risk group (*n* = 7) included those with both unfavorable CTC counts and CTC-clusters. Note that the one patient with one CTC that was a 2-cell cluster was excluded from this analysis because this subgroup only had one subject.

**Figure 2:**
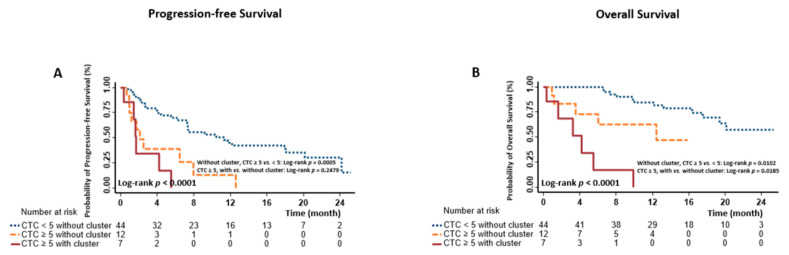
Kaplan-Meier estimates of the probability of progression-free survival (PFS) and overall survival (OS) in mCRPC. Survival differences were compared among those with <5 CTCs and 0 CTC-cluster, with ≥5 CTCs and 0 CTC-cluster, and with ≥5 CTCs and ≥1 CTC-clusters ((**A**) for PFS and (**B**) for OS).

Patients in the low-risk group had the longest survival time, whereas those in the high-risk group had the shortest survival time (median survival time 11.3 vs. 2.1 vs. 1.7 months for low-, medium-, and high-risk group, respectively, log-rank *p* < 0.0001 for PFS analysis; not reached vs. 12.4 vs. 4.2 months for low-, medium-, and high-risk group, respectively, log-rank *p* < 0.0001 for OS analysis) (Figure 2A,B and Table 2). Among patients with unfavorable CTCs, we further compared the survival difference between those with CTC-clusters and those without, and found a significant difference in OS (*p* = 0.0185), suggesting improved prognostic stratification using CTC-clusters. In the univariate Cox analyses, compared with the patients in the low-risk group, those in medium- and high-risk groups had a 3.53-fold (HR 3.53, 95% CI 1.66–7.51) and 6.30-fold (HR 6.30, 95% CI 2.40–16.53) increased risk for progression, as well as a 3.96-fold (HR 3.96, 95% CI 1.29–12.21) and 21.53-fold (HR 21.53, 95% CI 6.64–69.85) increased risk for death (Table 2). mCRPC patients with both unfavorable CTCs and CTC-clusters still had the highest risk for death (adjusted HR 19.84, *p* = 0.0072) in the multivariate Cox analyses (Table 2).

### 2.5. Prognostic Stratification Using Longitudinal CTCs and CTC-Clusters

In the joint analyses of CTCs and CTC-clusters described above, we found that baseline CTC-clusters could further stratify patients with unfavorable baseline CTCs into different risk groups. However, using measurements with only one time point may underestimate prognostic values. In comparison, using longitudinal data obtained from repeated measurements of each individual over time is an effective approach to improve prediction power [24,28].

To confirm the additional prognostic value of CTC-clusters, and to clarify whether the non-significant finding in PFS analyses was due to relatively small sample size, we evaluated the associations of longitudinal changes in CTCs and CTC-clusters with clinical outcomes, using the Cox proportional hazards model with time-dependent covariates. The time-dependent CTC- and cluster-related variables, including risk groups, were re-defined for every patient at each time point of blood draw from baseline to first progression or death [24]. In total, 227 longitudinally collected samples (median 3 samples, range 1–10 samples for each patient) with both CTC and CTC-cluster enumeration results were included in this analysis. Among these samples, 76 (33.5%) had ≥5 CTCs, and 27 (11.9%) had CTC-clusters (Table 3), of which 14 (51.9%) comprised one or more 2-cell clusters. Similar to what we had conducted in the analyses using baseline samples, we excluded one sample with one CTC that was a 2-cell cluster (*n* = 1) from joint analyses. As shown in Table 3, we found that high CTC counts (≥5) were significantly associated with unfavorable outcomes (*p* < 0.0001). The association between the presence of CTC-clusters and outcomes remained significant in OS-related analyses, even after adjustment for covariates (*p* < 0.05). In the joint analysis using longitudinal CTCs and CTC-clusters at each time point, we found that the death risk for patients with both unfavorable CTCs and CTC-clusters almost doubled, as compared to those with unfavorable CTCs but without a CTC-cluster (using patients without CTC as the reference group, adjusted HR 48.17 vs. 28.15; using patients with 1–4 CTCs as the reference group, adjusted HR 16.79 vs. 9.81). These results from longitudinal analyses were consistent with the baseline analyses and further confirmed that CTC-clusters conferred additional prognostic information to CTC enumeration alone and improved prognostic stratification in patients with unfavorable CTCs.

**Figure 3:**
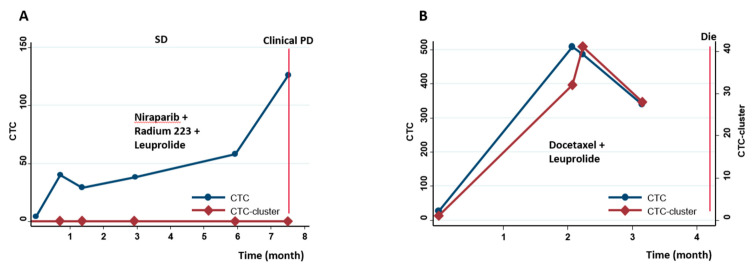
Longitudinal monitoring of CTCs and CTC-clusters in individual patients. (**A**) A patient had high CTC counts but without any CTC-cluster; (**B**) A patient had both high CTC counts and CTC-clusters.

We also plotted the dynamic changes of CTCs and CTC-clusters of individual patients. Figure 3A shows a patient who had persistently increased CTC counts after enrollment, but no CTC-cluster was identified during repeated measurements. This patient had a radiologically-confirmed stable disease for >7 months and then experienced clinical progression. In comparison, another patient who had both high CTC counts and CTC-clusters in each detection died soon (Figure 3B), which further indicated the possible additional prognostic information from CTC-clusters.

## 3. Discussion

The prognostic values of CTC-clusters have been reported, but mostly only in breast cancer. Despite a seminal study on CTC-clusters in PCa [22], no study has comprehensively evaluated the relevance of CTC-clusters in mCRPC, the most aggressive and deadly form of PCa. In the present study, we investigated the associations of CTCs and CTC-clusters with the survival of mCRPC patients, individually and jointly. As expected, we confirmed the associations of CTC counts with PFS and OS in patients with mCRPC in both baseline and longitudinal analyses. Our results also suggested that the presence of CTC-clusters alone had prognostic value and that CTC-clusters may further improve CTC-based prognostic stratification in mCRPC patients.

CTC-clusters are derived from multicellular groups of tumor cells that are held together through plakoglobin-dependent intercellular adhesion [22]. Clusters might be pre-formed before entering the blood or aggregate within the bloodstream [29]. Clusters are infrequent in peripheral blood, particularly in patients with early-stage cancer, presumably due to being trapped in narrow blood vessels; however, a recent study demonstrated that over 90% of clusters containing up to 20 cells successfully traversed 5- to 10-µm constrictions even in whole blood [21,30]. CTC-clusters have been identified in the circulation of patients with solid tumors including PCa [22,25,26,27,31,32]. Cohesive-clusters of human PCa cells were observed at multiple stages of dissemination as well [33]. In the present study, we identified CTC-clusters in 12.5% baseline samples from mCRPC patients and in 11.9% longitudinally collected samples. The detection rate of CTC-clusters in our study was higher than a report in PCa (9.4%) [22], but lower than two reports in mCRPC (17.1% and 50%) [26,27]. A study in prostate cancer recently showed that the CTC detection rate using the CellSearch system was the lowest when compared with the CellCollector and dual fluoro-EPISPOT assays [34]. Therefore, the varied detection rates between the studies of ours and others may be attributed to the heterogeneity of enrolled patients (PCa vs. mCRPC) and the use of different platforms for CTC enumeration. Compared with our previous enumeration results in patients with metastatic breast cancer (MBC) using the CellSearch platform [24], mCRPC patients had a lower detection rate of both CTCs (37.5% vs. 60.2% at baseline; 52.4% vs. 65.1% for longitudinal samples) and CTC-clusters (12.5% vs. 16.4% at baseline; 11.9% vs. 20% for longitudinal samples), likely due to the different molecular mechanisms underlying these two cancer types.

In vivo studies have shown that CTC-clusters had a 23- to 50-fold increase in metastatic potential compared to single CTCs in breast cancer [22]. Recent mechanism studies revealed that CTC-clusters have stem-like features, which facilitate metastasis initiation [29,35]. Intriguingly, the FDA-approved compounds that are able to dissociate CTC clusters lead to a decrease in metastasis formation [35]. So far, only a few studies have evaluated the prognostic value of CTC-clusters at the population level. In our previous studies in MBC patients, we found that CTC-clusters added additional prognostic values to CTC enumeration alone [23,24]. For the bone-predominant mCRPC, a non-invasive circulating biomarker, such as CTC-clusters, holds important potential clinical value for cancer prognostication.

The maintenance of cell-clusters, including cell-cell cohesive interactions, has been observed in the majority of invasive PCa [33,36]. Aceto et al. reported that the presence of CTC-clusters strongly correlated with a dramatically shorter OS of PCa patients [22]. Although a small study including 41 mCRPC patients failed to identify significant survival differences between those with and without CTC-clusters [26], a recent study conducted in the Japanese population found that CTC-clusters were independently associated with both PFS and OS in mCRPC patients treated with abiraterone or enzalutamide [27]. However, it was noted that, in that Japanese study, CTCs were not enumerated using the FDA-approved platform, and an extremely high detection rate (50%) of CTC-clusters was reported [27]. In our current study, we also observed that patients with CTC-clusters had significantly shorter survival compared to those without CTC-clusters (Table 2). Only the association between CTC-clusters and OS remained significant after adjusting conventional prognostic factors, such as performance status and treatments, indicating that a CTC-cluster was an independent prognostic factor for predicting long-term survival.

We then evaluated the role of CTC-clusters in prognostic stratification. After stratifying mCRPC patients with unfavorable CTC counts according to the presence or absence of CTC-clusters, we noted that OS differed significantly between these two groups (Figure 2B). Compared to the patients with <5 CTCs, those with both ≥5 CTCs and CTC-clusters had the highest risk for death (Table 2). We further conducted time-dependent analyses using 227 longitudinal samples, which confirmed the complementary prognostic values of CTC-clusters to CTC-enumeration alone (Table 3). However, unlike in breast cancer [24], we did not identify a correlation between the size of CTC-clusters and mCRPC survival. The majority of patients in the current study had only 2-cell CTC-clusters, which may reduce the power to identify such correlations, thus further assessment in larger future studies is needed.

The major strengths of this study include the focus on mCRPC that ensures a homogenous study population and the innovative use of time-dependent analyses of longitudinal samples with repeated measurements [24,37]. Our study also has several limitations. First, the results should be interpreted with caution because of the relatively small sample size. Future prospective studies with a large number of patients are warranted to confirm the findings in this study. Second, only <30% of patients had newly diagnosed mCRPC in our cohort. To address the potential confounding effects of treatments, we adjusted both previous and current treatments in the multivariate analyses (Table 2 and Table 3). Moreover, by conducting stratified analyses, we found that the results in the patients who received at least one line of previous therapy (Figure S2) were similar to that in the overall population. Third, we did not include LDH in the multivariate analyses because we failed to identify an association between LDH and clinical outcomes in the univariate analyses. LDH has been reported as an important prognostic factor for mCRPC [38]. The non-significant result in our study is possibly due to the high missing rate (>60%) of LDH among our patients. Fourth, we defined unfavorable CTC count using the cutoff of ≥5 CTCs. Although this cutoff is widely adopted, it may not be the optimal one according to the distribution of CTC counts in our dataset. Fifth, recent studies highlighted the role of molecular biomarkers in CTCs, for example, androgen receptor splice variants (particularly AR-V7) detected in CTCs as potential prognostic and predictive biomarkers for mCRPC [39,40,41]. Nevertheless, in the present study, we did not conduct genomic and molecular analyses of CTCs/CTC-clusters, which may further elucidate their clinical values in personalized therapeutic intervention.

## 4. Materials and Methods

### 4.1. Study Population

We recruited men with mCRPC who visited the Sidney Kimmel Cancer Center at Thomas Jefferson University Hospital from March 2018. The enrolled patients had histologically confirmed prostate adenocarcinoma, a progressive disease despite castration levels of serum testosterone (<50 ng/dL), and radiographic metastases according to computed tomography (CT) or technetium-99 bone scan. Patients were excluded if they concurrently had other primary tumors. Demographic data (e.g., age, race), clinical data (e.g., ECOG performance status, treatments), and laboratory data (e.g., PSA, HGB, ALP, ALB, LDH) were collected by reviewing medical charts. Blood samples were collected from each patient at baseline before initiation of a new therapy and at follow-up visits (approximately every 6–8 weeks) for CTC and CTC-cluster enumerations. Assessments of CTC/CTC-cluster, PSA, and tumor lesions were repeated. Follow-up imaging tests were conducted following the PCWG3 guideline [42]. This research has been approved by the Institutional Review Board of Thomas Jefferson University on 10 September 2015 (ethic code: #13D.507). All patients provided written informed consent to participate in this study.

### 4.2. CTC and CTC-Cluster Enumeration

Approximately 8–10 mL of peripheral blood was drawn into a 10 mL evacuated blood draw tube (CellSave tube, MENARINI Silicon biosystems, Huntington Valley, Pennsylvania, USA), maintained at room temperature, and processed within 96 h of collection. CTC and CTC-cluster enumerations were conducted using the CellSearch System (MENARINI Silicon biosystems), which consists of the CellTracks Autoprep and the CellSearch CTC kit to immunomagnetically enrich cells expressing the epithelial cell adhesion molecule (EpCAM) and fluorescently label nuclei (DAPI), leukocytes with monoclonal antibodies specific for leukocytes (CD45), and epithelial cells (phycoerythrin-conjugated cytokeratins CK-8,18,19). CTCs were defined as nucleated cells lacking CD45 and expressing cytokeratin (CK+/DAPI+/CD45−) [12]. CTC-clusters were defined as an aggregation of two or more individual CTCs containing distinct nuclei and intact cytoplasm membranes [23,24].

### 4.3. Statistical Analyses

Clinical outcomes analyzed in this study included PFS and OS. PFS was defined as the time from the date of baseline blood draw to the date of radiologic progression (on CT scan: ≥20% enlargement in sum diameter of target lesions (Response Evaluation Criteria in Solid Tumors) [43]; on bone scan: ≥2 new bone lesions not caused by flare), symptomatic progression (worsening disease-related symptoms or new cancer-related complications), or death, whichever occurred first [41]. OS was defined as the time from the date of baseline blood draw to the date of death from any cause. The patients without an endpoint event at the last follow-up visit were censored. Survival curves were plotted using the Kaplan-Meier method, and survival differences were compared using the log-rank test. Associations of CTC and CTC-cluster counts individually and jointly with PFS or OS were evaluated using HRs with 95% CIs by univariate and multivariate Cox proportional hazards models. The associations of longitudinal CTCs and CTC-clusters with clinical outcomes were analyzed as previously [24]. All statistical analyses were conducted using STATA (Version 11.0, STATA Corp., College Station, TX, USA) and R (package “survival”, Version 4.0.2, R Foundation for Statistical Computing, Vienna, Austria. URL http://www.r-project.org) software packages. Two-sided *p* values of <0.05 were considered to be of statistical significance.

## 5. Conclusions

To our best knowledge, this is the first comprehensive analysis of the role of prognosis stratification by CTC-clusters in mCRPC patients. Our findings suggest the potential of combing CTC and CTC-clusters as non-invasive means to monitor progression and predict survival in mCRPC and build a premise for in-depth genomic and molecular analyses of CTCs and CTC-clusters.

## Figures and Tables

**Table 1:** Patient characteristics ^†^.

Variables	*n* (%)
Age (year), median (range)	71.8 (53.0–93.0)
Race	
White	49 (76.6)
Black	12 (18.8)
Other	3 (4.7)
ISUP Grade at Diagnosis	
1	3 (4.7)
2	5 (7.8)
3	5 (7.8)
4	11 (17.2)
5	32 (50.0)
Unknown	8 (12.5)
ECOG Performance Status	
0	28 (43.8)
1	25 (39.1)
2	8 (12.5)
3	2 (3.1)
Unknown	1 (1.6)
Bone Metastasis	
No	4 (6.3)
Yes	60 (93.8)
Visceral Metastasis	
No	51 (79.7)
Yes	13 (20.3)
Previously Treated by AR Signaling Inhibitors *	
No	29 (45.3)
Yes	35 (54.7)
Previous Chemotherapy *	
No	54 (84.4)
Yes	10 (15.6)
AR Signaling Inhibitors After Baseline Blood Draw	
No	20 (31.3)
Yes	44 (68.8)
Cytotoxic Therapy After Baseline Blood Draw	
No	48 (75.0)
Yes	16 (25.0)
Prostate-specific antigen (ng/mL), median (range)	9.5 (0.1–3256.0)
Hemoglobin (g/dL), median (range)	12.0 (7.4–15.9)
Alkaline phosphatase (IU/L), median (range)	88.5 (31.0–1709.0)
Albumin (g/dL), median (range)	4.1 (2.6–4.7)
Lactate dehydrogenase (IU/L), median (range) ^#^	216 (149–560)

ISUP: International Society of Urological Pathology; ECOG: Eastern Cooperative Oncology Group; AR: androgen receptor. ^†^: characteristics at the first time of biomarker measurement (baseline), otherwise specifically described; *: previous treatments received from metastatic diagnose to the baseline sample collection; ^#^: data were available from 22 patients.

**Table 2:** Associations of baseline CTC and CTC-cluster with clinical outcomes.

Variables	Event/Total	MST (mo)	Univariate Analyses		Multivariate Analyses *	
			HR (95% CI)	*p*	HR (95% CI)	*p*
*Association With PFS*						
CTC						
<5	29/45	10.5	1.00		1.00	
≥5	16/19	2.1	4.03 (2.09–7.77)	<0.0001	3.01 (1.30–6.95)	0.0101
CTC-Cluster						
0	38/56	7.5	1.00		1.00	
≥1	7/8	1.7	4.33 (1.81–10.37)	0.0010	2.36 (0.70–7.95)	0.1666
Risk Group						
CTC < 5 without CTC-cluster	28/44	11.3	1.00		1.00	
CTC ≥ 5 without CTC-cluster	10/12	2.1	3.53 (1.66–7.51)	0.0011	3.51 (1.38–8.89)	0.0082
CTC ≥ 5 with CTC-cluster	6/7	1.7	6.30 (2.40–16.53)	0.0002	2.57 (0.62–10.59)	0.1909
*Association With OS*						
CTC						
<5	12/45	NR	1.00		1.00	
≥5	11/19	6.0	6.90 (2.72–17.47)	<0.0001	4.66 (1.38–15.74)	0.0134
CTC-Cluster						
0	17/56	NR	1.00		1.00	
≥1	6/8	4.2	6.58 (2.50–17.33)	0.0001	5.52 (1.18–25.79)	0.0299
Risk Group						
CTC < 5 without CTC-cluster	12/44	NR	1.00		1.00	
CTC ≥ 5 without CTC-cluster	5/12	12.4	3.96 (1.29–12.21)	0.0165	1.42 (0.32–6.33)	0.6497
CTC ≥ 5 with CTC-cluster	6/7	4.2	21.53 (6.64–69.85)	<0.0001	19.84 (2.24–175.32)	0.0072

CTC: circulating tumor cell; PFS: progression free survival; OS: overall survival; MST: median survival time; HR: hazard ratio; CI: confidence interval; NR: not reached. *: Adjusted for performance status, previous therapies, treatments after blood draw, prostate-specific antigen, hemoglobin, alkaline phosphatase, and albumin. Two different clinical endpoints (PFS and OS) were observed and analyzed. The italic words are explanation of the following results.

**Table 3:** Associations Time-dependent analysis of CTCs and CTC-clusters with clinical outcomes.

Variables	Univariate Analyses		Multivariate Analyses *	
	HR (95% CI)	*p*	HR (95% CI)	*p*
*Association with PFS*				
CTC				
0	1.00		1.00	
1–4	4.25 (1.98–9.13)	0.0002	4.98 (2.11–11.74)	0.0002
≥5	9.72 (4.38–21.58)	<0.0001	8.07 (2.91–22.41)	<0.0001
CTC-Cluster				
0	1.00		1.00	
≥1	4.97 (2.15–11.50)	0.0002	2.59 (0.85–7.88)	0.0947
Risk Group				
0 CTC	1.00		1.00	
1–4 CTCs without CTC-cluster	4.09 (1.86–8.97)	0.0004	4.55 (1.85–11.18)	0.0010
≥5 CTCs without CTC-cluster	8.47 (3.41–21.04)	<0.0001	8.83 (2.79–27.92)	0.0002
≥5 CTCs with CTC-cluster	12.11 (4.47–32.80)	<0.0001	7.59 (1.82–31.73)	0.0055
1–4 CTCs without CTC-cluster	1.00		1.00	
≥5 CTCs without CTC-cluster	2.07 (0.77–5.54)	0.1471	1.94 (0.56–6.72)	0.2959
≥5 CTCs with CTC-cluster	2.96 (1.02–8.57)	0.0451	1.67 (0.42–6.56)	0.4638
*Association with OS*				
CTC				
0	1.00		1.00	
1–4	3.60 (0.72–17.90)	0.1180	2.51 (0.38–16.43)	0.3378
≥5	32.84 (8.42–128.20)	<0.0001	28.47 (3.85–210.36)	0.0010
CTC-Cluster				
0	1.00		1.00	
≥1	9.16 (3.64–23.04)	<0.0001	3.90 (1.07–14.20)	0.0387
Risk Group				
0 CTC	1.00		1.00	
1–4 CTCs without CTC-cluster	3.66 (0.73–18.20)	0.1130	2.87 (0.42–19.77)	0.2847
≥5 CTCs without CTC-cluster	26.56 (6.35–111.00)	<0.0001	28.15 (3.62–218.61)	0.0014
≥5 CTCs with CTC-cluster	46.35 (10.65–201.80)	<0.0001	48.17 (4.10–566.62)	0.0021
1–4 CTCs without CTC-cluster	1.00		1.00	
≥5 CTCs without CTC-cluster	7.26 (1.70–31.01)	0.0074	9.81 (1.70–56.62)	0.0107
≥5 CTCs with CTC-cluster	12.67 (2.83–56.84)	0.0009	16.79 (2.30–122.73)	0.0054

CTC: circulating tumor cell; PFS: progression free survival; OS: overall survival; HR: hazard ratio; CI: confidence interval. *: Adjusted for performance status, previous therapies, treatments after blood draw, prostate-specific antigen, hemoglobin, alkaline phosphatase, and albumin. Two different clinical endpoints (PFS and OS) were observed and analyzed. The italic words are explanation of the following results.

## Data Availability

The datasets used and/or analyzed during the current study are available from the corresponding author on reasonable request.

## Supplementary Materials

The following are available online at https://www.mdpi.com/2072-6694/13/2/268/s1, Figure S1. CTC and CTC-clusters images from 7.5 mL of blood of mCRPC patients analyzed by the CellSearch platform, Figure S2. Kaplan-Meier estimates of probability of progression-free survival (PFS) and overall survival (OS) in mCRPC patients who were previously treated, Table S1: Enumeration results of CTCs and CTC-clusters at baseline, Table S2: Univariate analyses of associations with clinical outcomes.
