# Supplementary material for: Improved Prognostic Stratification Using Circulating Tumor Cell Clusters in Patients with Metastatic Castration-Resistant Prostate Cancer
Source: Cancers (Basel). 2021 Jan 13;13(2):268. doi: 10.3390/cancers13020268 (PMC7828213; doi:10.3390/cancers13020268)
Supplement: Supplementary file 1 [file cancers-13-00268-s001.pdf]

cancers

Improved Prognostic Stratification Using Circulating Tumor Cell Clusters in Patients with Metastatic Castration-Resistant Prostate Cancer

Chun Wang, Zhenchao Zhang, Weelic Chong, Rui Luo, Ronald E. Myers, Jian Gu, Jianqing Lin, Qiang Wei, Bingshan Li, Timothy R. Rebbeck, Grace Lu-Yao, William K. Kelly and Hushan Yang

Citation: Wang, C.; Zhang, Z.; Chong, W.; Luo, R.; Myers, R.E.; Gu, J.; Lin, J.; Wei, Q.; Li, B.; Rebbeck, T.R.; et al. Improved Prognostic Stratification Using Circulating Tumor Cell Clusters in Patients with Metastatic Castration-Resistant Prostate Cancer. *Cancers* **2021**, *13*, 268. https://doi.org/10.3390/cancers13020268

Received: 12 November 2020
Accepted: 7 January 2021
Published: 13 January 2021

Publisher's Note: MDPI stays neutral with regard to jurisdictional claims in published maps and institutional affiliations.

Copyright: © 2021 by the authors. Licensee MDPI, Basel, Switzerland. This article is an open access article distributed under the terms and conditions of the Creative Commons Attribution (CC BY) license (http://creativecommons.org/licenses/by/4.0/).

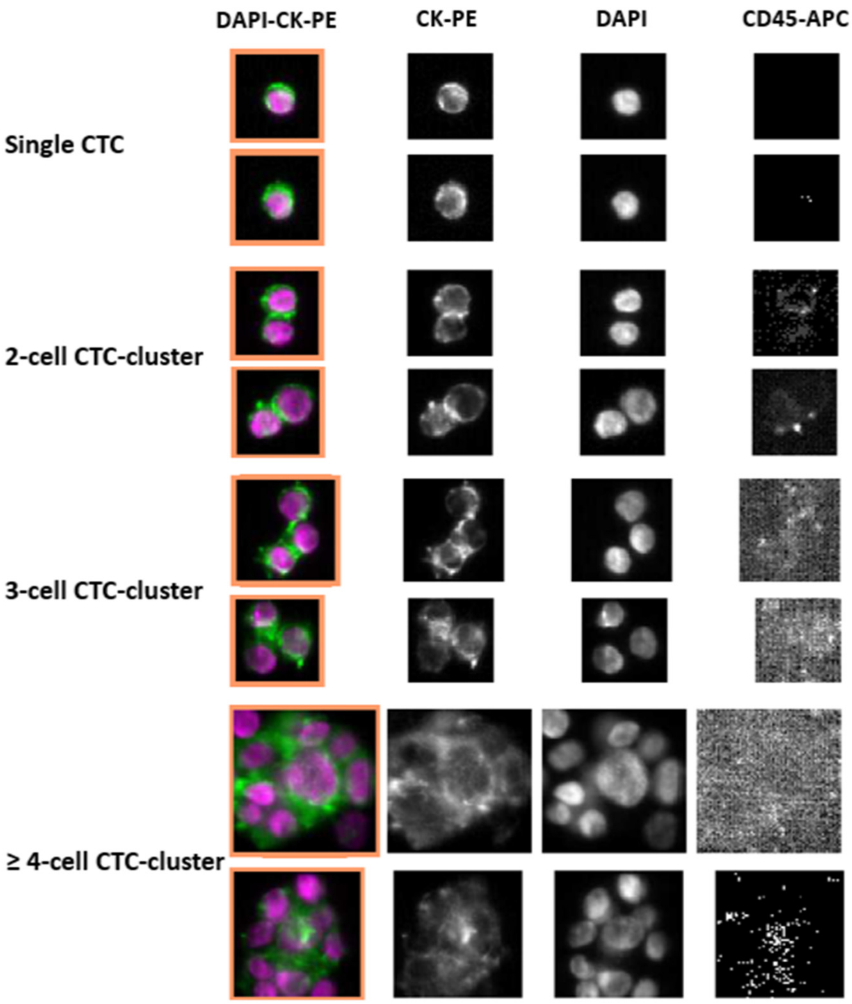

Figure S1. CTC and CTC-clusters images from 7.5 mL of blood of mCRPC patients analyzed by the CellSearch platform. CTCs and CTC-clusters were identified by the following criteria: CK-PE+ (phycoerythrin (PE)-conjugated cytokeratins), DAPI+ (Nucleic acid dye), and CD45− (Allophycocyanin (APC)-conjugated anti-CD45).

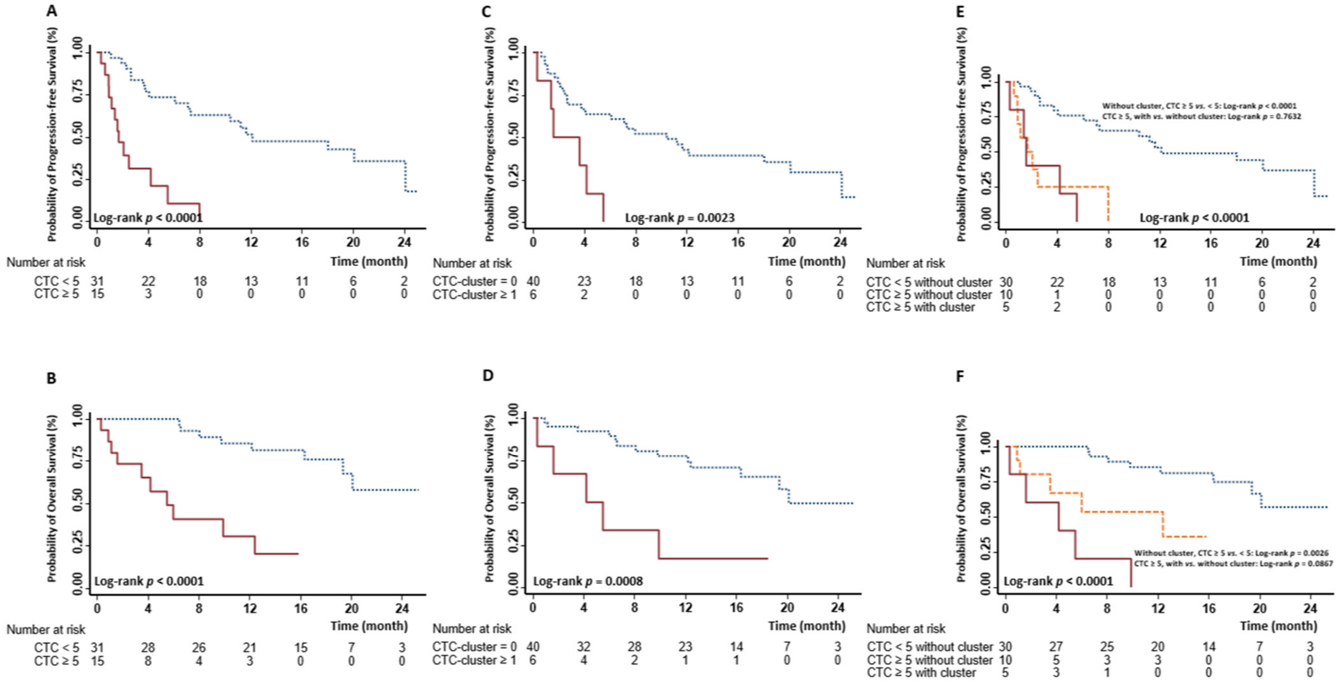

Figure S2. Kaplan-Meier estimates of the probability of progression-free survival (PFS) and overall survival (OS) in mCRPC patients who were previously treated. Risk groups were defined by CTCs individually (**A** and **B**), or by CTC-clusters individually (**C** and **D**), or by the combination of CTCs and CTC-clusters (**E** and **F**).

Table S1. Enumeration results of CTCs and CTC-clusters at baseline.

ID	CTC	CTC-Cluster	2-Cell Cluster	3-Cell Cluster	≥ 4-Cell Cluster
1	12	1	1	0	0
2	0	0	0	0	0
3	0	0	0	0	0
4	0	0	0	0	0
5	0	0	0	0	0
6	18	0	0	0	0
7	0	0	0	0	0
8	4	0	0	0	0
9	45	1	1	0	0
10	0	0	0	0	0
11	0	0	0	0	0
12	0	0	0	0	0
13	0	0	0	0	0
14	0	0	0	0	0
15	26	1	1	0	0
16	0	0	0	0	0
17	0	0	0	0	0
18	28	0	0	0	0
19	128	0	0	0	0
20	0	0	0	0	0
21	1	1	1	0	0
22	0	0	0	0	0
23	0	0	0	0	0
24	0	0	0	0	0
25	0	0	0	0	0
26	0	0	0	0	0
27	228	24	13	9	2
28	0	0	0	0	0
29	0	0	0	0	0
30	6	0	0	0	0
31	0	0	0	0	0
32	37	3	2	0	1
33	0	0	0	0	0
34	0	0	0	0	0
35	0	0	0	0	0
36	2	0	0	0	0
37	0	0	0	0	0
38	0	0	0	0	0
39	0	0	0	0	0
40	0	0	0	0	0
41	0	0	0	0	0
42	0	0	0	0	0
43	0	0	0	0	0
44	5	0	0	0	0
45	6	0	0	0	0
46	5	0	0	0	0

47	0	0	0	0	0
48	0	0	0	0	0
49	0	0	0	0	0
50	0	0	0	0	0
51	1	0	0	0	0
52	4	0	0	0	0
53	5	0	0	0	0
54	0	0	0	0	0
55	6	0	0	0	0
56	0	0	0	0	0
57	15	0	0	0	0
58	30	0	0	0	0
59	0	0	0	0	0
60	0	0	0	0	0
61	37	1	1	0	0
62	17	0	0	0	0
63	225	14	11	2	1
64	0	0	0	0	0

CTC: circulating tumor cell; CTC-cluster: circulating tumor cell cluster.

Table S2. Univariate analyses of associations with clinical outcomes.

**Variables**	***n***	**Progression-Free Survival**		**Overall Survival**	
		**HR (95% CI)**	***p***	**HR (95% CI)**	***p***
Age (year)	64	1.00 (0.96–1.03)	0.7979	0.99 (0.95–1.04)	0.7953
< 71.75	32	1.00		1.00	
≥ 71.75	32	0.85 (0.47–1.53)	0.5914	0.97 (0.43–2.21)	0.9451
Race					
White	49	1.00		1.00	
Black	12	0.96 (0.46–2.02)	0.9109	0.50 (0.15–1.68)	0.2612
Other	3	1.32 (0.32–5.53)	0.7016	-	0.9903
ISUP grade at diagnosis					
1–3	13	1.00		1.00	
4	11	1.02 (0.39–2.64)	0.9719	0.66 (0.16–2.75)	0.5644
5	32	0.96 (0.44–2.10)	0.9247	0.93 (0.33–2.64)	0.8867
ECOG performance status					
0	28	1.00		1.00	
1	25	0.98 (0.51–1.89)	0.9575	3.56 (1.14–11.05)	0.0283
≥ 2	10	1.91 (0.83–4.40)	0.1293	14.53 (4.07–51.85)	< 0.0001
Bone metastasis					
No	4	1.00		1.00	
Yes	60	4.47 (0.60–33.37)	0.1444	-	0.9926
Visceral metastasis					
No	51	1.00		1.00	
Yes	13	0.71 (0.33–1.52)	0.3723	1.17 (0.46–3.01)	0.7437
Previously treated by ARSi					
No	29	1.00		1.00	
Yes	35	1.15 (0.64–2.08)	0.6344	1.74 (0.75–4.04)	0.1955
Previous chemotherapy					
No	54	1.00		1.00	
Yes	10	2.76 (1.30–5.84)	0.0080	7.81 (3.02–20.16)	< 0.0001

ARSi after baseline blood draw					
No	20	1.00		1.00	
Yes	44	0.64 (0.34–1.20)	0.1603	0.32 (0.14–0.76)	0.0093
Chemotherapy after baseline blood draw					
No	48	1.00		1.00	
Yes	16	4.80 (2.39–9.65)	< 0.0001	3.71 (1.61–8.52)	0.0020
Prostate–specific antigen (ng/mL)	64	1.22 (1.12–1.33) *	< 0.0001	1.36 (1.18–1.56) *	< 0.0001
< 9.45	32	1.00		1.00	
≥ 9.45	32	3.71 (1.94–7.09)	< 0.0001	5.48 (2.07–14.49)	0.0006
Hemoglobin (g/dL)	64	0.14 (0.04–0.54) *	0.0043	0.02 (0.004–0.11) *	< 0.0001
< 11.95	32	1.00		1.00	
≥ 11.95	32	0.57 (0.31–1.03)	0.0641	0.42 (0.18–0.98)	0.0442
Alkaline phosphatase (IU/L)	64	1.64 (1.20–2.25) *	0.0021	3.20 (2.04–5.00) *	< 0.0001
< 88.5	32	1.00		1.00	
≥ 88.5	32	1.37 (0.76–2.49)	0.2944	2.48 (1.05–5.88)	0.0391
Albumin (g/dL)	64	0.07 (0.01–0.46) *	0.0053	0.004 (0–0.07) *	< 0.0001
< 4.1	29	1.00		1.00	
≥ 4.1	35	0.59 (0.33–1.06)	0.0754	0.30 (0.12–0.74)	0.0084
Lactate dehydrogenase (IU/L)	22	2.66 (1.02–6.99) *	0.0466	25.55 (2.18–299.23) *	0.0099
< 216	10	1.00		1.00	
≥ 216	12	1.19 (0.46–3.09)	0.7150	5.43 (0.63–46.52)	0.1226

ISUP: International Society of Urological Pathology; ECOG: Eastern Cooperative Oncology Group; ARSi: androgen receptor signaling inhibitors; HR: hazard ratio; CI: confidence interval. *After log transformation.
